# Asymptomatic Pembrolizumab-Associated Hypertriglyceridemia

**DOI:** 10.7759/cureus.110286

**Published:** 2026-06-05

**Authors:** Lauren S Yeager, Ralph G Zinner, Zhonglin Hao

**Affiliations:** 1 Division of Medical Oncology, University of Kentucky Markey Cancer Center, Lexington, USA

**Keywords:** asymptomatic hypertriglyceridemia, immunotherapy hypertriglyceridemia, immunotherapy-related adverse events, metastatic non-small cell lung cancer, pembrolizumab-associated hypertriglyceridemia, pembrolizumab toxicity, rare immunotherapy adverse event

## Abstract

Pembrolizumab is an immune checkpoint inhibitor that is widely used in the treatment of solid and hematological malignancies but is associated with immune-related adverse events. While the management of pembrolizumab-associated symptomatic hypertriglyceridemia associated with pancreatitis has been described, there is a lack of guidance for the treatment of pembrolizumab-associated asymptomatic hypertriglyceridemia. Here, we report a rare adverse event of pembrolizumab-associated asymptomatic hypertriglyceridemia after nine doses of pembrolizumab. The patient’s lipid panel revealed grade 4 hypertriglyceridemia and hypercholesterolemia. Treatment was held, and the patient was treated with lipid-lowering therapies with improvement. Upon re-challenge, the grade 4 elevations returned while remaining asymptomatic. This case highlights a rare immune-related adverse event of asymptomatic hypertriglyceridemia. It underlines the importance of routine monitoring for adverse events with pembrolizumab, even in asymptomatic cases.

## Introduction

Pembrolizumab is a monoclonal antibody that binds to programmed death protein-1 (PD-1) on the surface of T cells, which prevents PD-1 from interacting with programmed cell death ligand 1 (PD-L1) and, in turn, allows T cells to have an anti-tumor immune response. Immune checkpoint inhibitors (ICI) are widely used in solid and hematological malignancies but are associated with immune-related adverse events (IrAEs). Common IrAEs include rash, colitis, and hypothyroidism [1]. We report a case of hypertriglyceridemia (HTG) associated with pembrolizumab in a patient with metastatic non-small-cell lung cancer (NSCLC). Existing literature gives guidance for immunotherapy-associated HTG cases associated with pancreatitis, but this is the first case report to our knowledge that describes a case without acute complications. HTG can have many causes, which include but are not limited to medications and hypothyroidism. Previous reports treated immunotherapy-associated pancreatitis with continuous insulin and apheresis [2,3]. Management of HTG without an acute complication involves holding treatment, monitoring for the development of pancreatitis or other acute complications, and prompt titration of lipid-lowering medications. Understanding asymptomatic pembrolizumab-associated HTG is clinically important in order to avoid treatment delays in cancer patients.

## Case presentation

Informed consent and permission to publish were obtained from the family. A 69-year-old woman with metastatic lung adenocarcinoma with Her2 exon 20 insertion presented for cycle five pembrolizumab maintenance following four cycles of carboplatin, pemetrexed, and pembrolizumab. Baseline lipid panel prior to treatment start showed mild elevations with triglycerides (TG) 214 mg/dL (Common Terminology Criteria for Adverse Events (CTCAE) grade 1) and total cholesterol (TC) 254 mg/dL (CTCAE grade 1) [4]. She reported labs drawn with her primary care physician, which showed TG 872 mg/dL (CTCAE grade 3) and TC above the assay's reportable range. She had received nine doses of pembrolizumab 200 mg prior. She came fasted to the appointment to repeat labs. Labs revealed TG 1,343 mg/dL (CTCAE grade 4) and TC 738 mg/dL (CTCAE grade 4). Thyroid-stimulating hormone (TSH) was also elevated to 112 mIU/L. She had a pre-existing pembrolizumab-related thyroid dysfunction starting with cycle three pembrolizumab maintenance six weeks prior, shown by a TSH of 63.3 mIU/L. She was prescribed levothyroxine 75 mcg daily at the time, but at the time of the elevated lipid panel, she admitted to never starting the levothyroxine. She had no signs or symptoms of acute pancreatitis, and amylase and lipase were both within normal range. Pembrolizumab was held, and she was started on fenofibrate 54 mg daily, rosuvastatin 20 mg nightly, and levothyroxine 88 mcg daily. She was not admitted due to the patient wishing to avoid the emergency department and having no signs of acute pancreatitis, but she was given strict instructions on when to go to the emergency department. Repeat fasted lipid panel showed improvement with TG 622 mg/dL (CTCAE grade 3) and TC 539 mg/dL (CTCAE grade 4). She continued to be asymptomatic with normal amylase and lipase levels, so close observation was continued with precautions for calling the clinic and going to the emergency department. Fenofibrate was increased to 108 mg daily, and rosuvastatin was continued at 20 mg daily. TSH decreased to 72.9 mIU/L, and levothyroxine was increased to 100 mcg daily.

Two months after holding pembrolizumab, it was resumed due to improvement in labs and concern from the oncology team and patient about her metastatic cancer being untreated. The risk of her lipids worsening was discussed in depth with the patient, but due to being asymptomatic, the patient wishing to resume treatment, and improvement in lipids, the decision was made to re-challenge pembrolizumab. TG was 422 mg/dL (CTCAE grade 2) and TC was 455 mg/dL (CTCAE grade 3) on the day of resumption. Lipid panel and TSH were planned to be monitored every three weeks prior to pembrolizumab treatment. There was a delay in the next cycle and lab check by three weeks due to missed appointments. At the next lipid panel check, TG was 1,456 mg/dL (CTCAE grade 4) and TC was 630 mg/dL (CTCAE grade 4). TSH was 147 mIU/L, and levothyroxine was increased to 150 mcg daily. Amylase and lipase were within normal limits, and the patient had no signs of pancreatitis. Pembrolizumab was permanently discontinued due to recurrent grade 4 HTG. Labs were planned to be monitored two weeks after but were not obtained due to a missed appointment. The patient was counseled on the signs of pancreatitis, which would warrant immediate evaluation. She also received a follow-up phone call one week later to confirm she remained asymptomatic. Withholding treatment and continuing maximum doses of lipid-lowering agents, the lipid panel improved to grade 2 elevations.

Table 1 shows the full timeline of lipid panel values and medication interventions. TSH was 149 mIU/L on the day of permanent discontinuation, but the lack of improvement was attributed to poor compliance with levothyroxine including multiple missed doses per week. The importance of compliance was emphasized. The Naranjo adverse drug reaction probability score for the patient was 5, as shown in Table 2, suggesting a probable adverse drug reaction [5]. The patient transitioned to surveillance after permanent pembrolizumab discontinuation and ultimately opted for best supportive care at a hospital closer to home upon progression four months later, so the follow-up laboratory values are unknown.

**Table 1:** Lipid Panel Timeline and Interventions CTCAE: Common Terminology Criteria for Adverse Events

Days from event	Triglycerides (reference range <150 mg/dL)	Triglycerides CTCAE grade	Total cholesterol (reference range <200 mg/dL)	Total cholesterol CTCAE grade	Medication intervention
Day 0	1,343 mg/dL	4	738 mg/dL	4	Hold pembrolizumab
					Begin fenofibrate 54 mg daily
					Begin rosuvastatin 20 mg daily
Day 21	622 mg/dL	3	539 mg/dL	4	Hold pembrolizumab
					Increase fenofibrate to 108 mg daily
					Continue rosuvastatin 20 mg daily
Day 43	422 mg/dL	2	455 mg/dL	3	Re-challenge pembrolizumab
					Continue fenofibrate 108 mg daily
					Continue rosuvastatin 20 mg daily
Day 86	1,456 mg/dL	4	630 mg/dL	4	Permanently discontinue pembrolizumab
					Increase fenofibrate to 145 mg daily
					Change rosuvastatin 40 mg daily to atorvastatin 80 mg daily
Day 128	396 mg/dL	2	422 mg/dL	3	Continue with surveillance
					Continue fenofibrate 145 mg daily
					Continue atorvastatin 80 mg daily

**Table 2:** Naranjo Algorithm [5]

Questions	Yes	No	Don’t know
Are there previous conclusive reports of this reaction?		0	
Did the adverse event appear after the drug was given?	2		
Did the adverse reaction improve when the drug was discontinued or a specific antagonist was administered?	1		
Did the adverse event reappear when the drug was re-administered?	2		
Are there alternative causes (other than the drug) that could on their own have caused the reaction?	-1		
Did the reaction reappear when a placebo was given?			0
Was the drug detected in blood (or other fluids) in concentrations known to be toxic?			0
Was the reaction more severe when the dose was increased or less severe when the dose was decreased?			0
Did the patient have a similar reaction to the drug or a related agent in the past?			0
Was the reaction confirmed by any objective evidence?	1		
	Total score: 5, probable adverse drug reaction		

## Discussion

Pembrolizumab’s package insert recommends withholding pembrolizumab for grade 3 or 4 endocrinopathies until clinically stable or permanently discontinue, depending on severity [1]. Of note, grade 3 or 4 amylase and lipase elevations are classified based on the presence of symptoms, whereas grade 3 or 4 TG and TC elevations are classified solely on the laboratory value. This patient experienced recurrent grade 4 HTG secondary to immunotherapy. Case reports of immunotherapy-associated HTG are limited to cases associated with pembrolizumab. Although not fully understood, the proposed mechanism of pembrolizumab-associated HTG is through the development of autoantibodies against GPIHBP1, preventing lipoprotein lipase (LPL) from reaching the capillary lumen. Consequently, this leads to low levels of LPL, impaired intravascular hydrolysis of TG, and severe HTG [6]. We were unable to draw a GPIHBP1 autoantibody lab due to it not being commercially available. Other cases report resolution of HTG after treatment with fenofibrate and a statin [7]. Fenofibrate is a peroxisome proliferator-activated receptor-alpha agonist, which upregulates the production of LPL and downregulates the production of apolipoprotein C-III to clear TG lipoproteins from the bloodstream [8]. Due to the proposed mechanism of pembrolizumab-associated HTG being associated with low levels of LPL, treatment with fenofibrate to upregulate LPL may counteract this mechanism. The Naranjo adverse drug reaction probability score of five suggests that the reaction followed a reasonable temporal sequence after a drug, followed a recognized response to the suspected drug, was confirmed by withdrawal but not by exposure to the drug, and could not be reasonably explained by the known characteristics of the patient’s clinical state. Alternative causes that could have contributed or caused this reaction include hypothyroidism, due to the hypothyroidism and HTG having similar timelines, and hypothyroidism is a known secondary cause of HTG. The pembrolizumab-related thyroid dysfunction had an onset of six weeks prior to the HTG and is a confounding factor. The patient’s poor compliance with levothyroxine makes the hypothyroidism course more difficult to interpret and correlate.

## Conclusions

More guidance is needed in terms of the appropriateness of re-challenge in patients with asymptomatic HTG. We recommend treatment with lipid-lowering agents, given no accompanying signs or symptoms of an acute complication such as pancreatitis. While immunotherapy-associated HTG is a rare IrAE with a paucity of data for treatment, our patient was successfully treated by holding immunotherapy and initiating lipid-lowering agents. The role of steroids for this IrAE is unknown. Future directions include a better understanding of the mechanism of pembrolizumab-associated HTG, the role of monitoring lipid levels during immunotherapy, and the safety of pembrolizumab re-challenge after asymptomatic HTG.
